# The Multifactorial Etiopathogeneses Interplay of Inflammatory Bowel Disease: An Overview

**DOI:** 10.3390/gidisord1010007

**Published:** 2018-10-18

**Authors:** Amosy E. M’Koma

**Affiliations:** 1 Meharry Medical College School of Medicine, Department of Biochemistry, Cancer Biology, Neuroscience and Pharmacology, Nashville, TN 37208, USA;; 2 Vanderbilt University School of Medicine, Department of Surgery, Colon and Rectal Surgery, Nashville, TN 37232, USA; 3 The American Society of Colon and Rectal Surgeons (ASCRS), Arlington Heights, IL 60005, USA; 4 The American Gastroenterological Association (AGA), Bethesda, MD 20814, USA; 5 Vanderbilt-Ingram Cancer Center (VICC), Vanderbilt University Medical Center, Nashville, TN 37232, USA

**Keywords:** gastrointestinal disorders, inflammatory bowel disease, ulcerative colitis, Crohn’s disease, indeterminate colitis, inflammation, rural lifestyle, urbanization, diet/nutrition, environment, intestinal microbiota, dysbiosis/dysbacteriosis), genetics, ethnicity, innate immune system, adaptive/acquired immune system

## Abstract

The gastrointestinal system where inflammatory bowel disease occurs is central to the immune system where the innate and the adaptive/acquired immune systems are balanced in interactions with gut microbes under homeostasis conditions. This article overviews the high-throughput research screening on multifactorial interplay between genetic risk factors, the intestinal microbiota, urbanization, modernization, Westernization, the environmental influences and immune responses in the etiopathogenesis of inflammatory bowel disease in humans. Inflammatory bowel disease is an expensive multifactorial debilitating disease that affects thousands new people annually worldwide with no known etiology or cure. The conservative therapeutics focus on the established pathology where the immune dysfunction and gut injury have already happened but do not preclude or delay the progression. Inflammatory bowel disease is evolving globally and has become a global emergence disease. It is largely known to be a disease in industrial-urbanized societies attributed to modernization and Westernized lifestyle associated with environmental factors to genetically susceptible individuals with determined failure to process certain commensal antigens. In the developing nations, increasing incidence and prevalence of inflammatory bowel disease (IBD) has been associated with rapid urbanization, modernization and Westernization of the population. In summary, there are identified multiple associations to host exposures potentiating the landscape risk hazards of inflammatory bowel disease trigger, that include: Western life-style and diet, host genetics, altered innate and/or acquired/adaptive host immune responses, early-life microbiota exposure, change in microbiome symbiotic relationship (dysbiosis/dysbacteriosis), pollution, changing hygiene status, socioeconomic status and several other environmental factors have long-standing effects/influence tolerance. The ongoing multipronged robotic studies on gut microbiota composition disparate patterns between the rural vs. urban locations may help elucidate and better understand the contribution of microbiome disciplines/ecology and evolutionary biology in potentially protecting against the development of inflammatory bowel disease.

## 1. Introduction

The “Colitides” also known as Inflammatory Bowel Disease (IBD), include ulcerative colitis (UC) and Crohn’s disease (CD), is intestinal disease that cause prolonged chronic relapsing and remitting inflammation of the digestive tract due to multifactorial interplay between genetic risk, the immune system, environmental exposures, and the intestinal microbiota in genetically susceptible individuals [1–3]. The aetiopathogenesis of UC and CD remains enigmatic [4,5]. The incidence of IBD is alarmingly evolving in pediatrics and young adults worldwide [4,5]. In the beginning of the 21st century, in some developed nations the incidence of IBD declined with a prevalence of as lower as up to 0.5% of the general population, while it has continued to rise in developing countries [6–8] as well as in some Western and developed countries [9–13].

IBD incidence and prevalence is evolving worldwide [14,15] and is now contemplated to be an emergence global disease [4]. The burden of IBD varies in different countries and locations, especially when compared between developing [16–31] and developed nations [32,33]. Data suggest that younger populations are more affected [6,34] and that the peak incidence of IBD occurs in children and adolescents [34,35]. It is estimated that 25 to 30 percent of cases diagnosed with CD and 20 percent of patients with UC present early in life and in most cases before the age of 20 years [35–37].

Rapid urbanization represents a major demographic shift and has been associated with an escalated incidence of several autoimmune diseases, including IBD [36,38–40]. In this long extensive literature search overview article we discuss: (i) *pathogenesis of IBD*, seeking to better understand accurately the aetiopathogenesis of IBD. (ii) *environmental factors*, building on the knowledge of how factors like diet, microbiota and psychological stress are reflected to play a role in IBD (iii) *preclinical human IBD mechanisms*, paying close attention to how IBD manifests in patients and ensuring research in the laboratory reflects this understanding, (iv) *novel technologies*, applying the latest multipronged innovations like non-invasive imaging and biosensors to IBD and (v) *pragmatic clinical research*, working in collaboration between basic scientist, clinical teams and patients to ultimately answer questions relevant to daily clinical practice and evaluate the effectiveness of current practices in diagnostics and treatments.

## 2. Methods

Performed literature review using multipronged search engine predetermined protocol in accordance with the quality assurance of reporting meta-analyses of observational contemplations (MOOSE) [41,42]. Preferred reporting items for review and meta-analysis protocols (PRISMA-P) was followed [43]. A comprehensive multipronged search of the “inflammatory bowel disease (IBD)” etiopathogenesis was carried out through 30 of June 2018 using Medical Literature Analysis, PubMed, and Retrieval System Online (MEDLINE), Current Nursing, Excerpta Medica database (EMBASE), and Allied Health Literature (CINAHL), Web of Science, the Cochrane library, and Google^®^ search engine. The following search terms were used: inflammatory bowel disease, indeterminate colitis, ulcerative colitis, Crohn’s disease, Crohn’s colitis, inflammation, etiology, pathogenesis, intestinal microbiota, genetic risk factors, environmental factors, diet, immune responses, Westernization culture, developed countries, urbanization, developing nations, diagnostics, and treatment. Subordinate and hand/manual searches of reference lists, other studies cross-indexed by reviews, authors, books, commentaries, and conference abstracts were also carry out. Published reports in language other than English, non-human studies and editorials were eliminated. Manuscript inclusions were based on the available supportive evidence for each particular detailed item of interest. Final, conclusive consensus was statistically evaluated with the *k-statistic* during the title and abstract reviews. As a result, titles were examined and divided into two sets when the value was ≥0.6; each was reviewed by the researcher. Assessable discrepancies were corrected, followed by other assessments of agreement when the value was <0.6.

## 3. Results

There were 51,671 publications identified in the review search of the possible etiopathogenesis of IBD (22,925 for UC, 27,536 for CD and 1210 for indeterminate colitis (IC). Of the 51,671 publications, 13,773 were duplicate publications and were excluded. Further, following a review of the abstract or title, additional 23,970 were removed because were found not to be relevant to the topic of etiopathogenesis, leaving 13,928 full-text articles. Of the 13,928 reviewed articles, 277 were further excluded because they were not English-language publications and/or non-human studies leaving 13,651 articles qualified for inclusion in this extensive summarized overview.

## 4. Etiopathogenesis of IBD

Inflammatory bowel disease has been understood to be idiopathic attributable to several possible effectuates that include genetic, gut microbiota, dysbiosis; pollution, hygiene, environmental, immune response, urbanization and dietary/nutritional factors [44,45]. These factors, and more, are discussed point by point below. The digestive system in which IBD takes place is central to both the innate and adaptive/acquired immune systems where are balanced in complex reciprocal influence with intestine luminal microbes under homeostasis premises [46]. In IBD however, etiopathogenesis has become better elucidated owing to scientific technology advances in biological genetic, environmental and immunology that normal homeostasis physiology is disrupted and uncontrolled intestinal inflammation is perpetuated [47,48]. Customary pattern of thought, Th1 cell have been observed to play a crucial role in etiopathogenesis related to the chronicity of intestinal inflammation, especially in CD, where Th2 cells have been thought to play an important influence in UC [46,49]. Recently, however, it has been reported that activation of Th17 cells and imbalance of Th17/regulatory T (Treg) cells are recognized to be a vital segmental component in the trigger and development of intestinal inflammation, such as IBD [50]. Since tumor necrosis factor (TNF)-α is a strong candidate and has been identified as a potential cytokine in IBD etiopathogenesis, the establishment of anti-TNF-α treatment has contributed towards the initiation of disease-remodeling drugs [50–52].

### 4.1. Genetic Risk Factors

Despite the fact that the exact cause of IBD still remains unclear, there are susceptibilities that has broadly been recognized to have a genetic component ground, a defective immune system [53–55], and environmental basis combined are thought to partly play role in the etiopathogenesis [56,57]. This is singled out by the development of IBD in immigrants to high-prevalence countries [58] and contention of IBD among monozygotic twins [59]. The significance of environmental components is also well recognized by a rising trend in the incidence and prevalence of IBD in countries undergoing rapid Westernization [4,16,60]. As mentioned, genetics is observed to play role as observed by the greater prevalence of IBD in Ashkenazim Jews with trace ancestry in northern-European Jewish groups than Sephardic Jewish population [61,62]. Analyzing data materials from 5685 Ashkenazi Jewish exomes, Rivas et al. [62] bring forth a systemic analysis of Ashkenazi Jewish enriched protein-coding alleles, which contribute to distinct in genetic risk to IBD of which are transmitted via autosomal recessive inheritance. Other similar such genome-wide scan studies are herewith in-depth discussed [63,64]. Genetic population isolates like the Ashkenazim, Jews who trace their ancestry to eleventh century central European Jewish groups [65], have hitherto made it possible the mapping of alleles to play a part in to human disorder predisposition [66–69]. The documented 2–4 fold enrichment of CD prevalence in the Ashkenazi Jewish population [70,71] prompted enthusiasm for the use of exome sequencing and genome-wide array studies to evaluate the degree to which bottle-neck-enriched protein-altering alleles and undeniably implicated common variants contribute an excess CD genetic risk to Ashkenazi Jewish [70]. Despite efforts in the advance in the mapping genes and alleles for physical injuries and/or disorders with increased prevalence in the Ashkenazi Jewish population, precise estimates of the risk-allele frequency and the carrier rate in the Ashkenazi Jewish population have unfortunately not yet been resolved to date [72]. In addition, the disproportion of immune responses to intestinal bacterial antigens is thought to play a critical role in the etiopathogenesis of IBD in genetically receptive host individuals [73].

The *CARD* family plays an important mechanistic role in innate immune response by the activation of nuclear factor-κB (NF-κB). Studies to determine the gene expression and enumeration of the protein-expressing cells of some members of the *CARD* family (*CARD9*, *CARD10*, *CARD11*, *CARD14* and *CARD15*) in patients with IBD vs. normal controls have demonstrated that the CARD9 and CARD10 gene expression was significantly elevated in UC as compared to CD. *CARD11* gene expression was significant decreased in UC than in CD patients while *CARD14* gene expression was significantly heightened in the group with active UC compared to non-inflamed controls. The down expression of *CARD14* gene was associated with a benign clinical course of UC, characterized by initial activity followed by long-term remission longer than 5 years. *CARD15* gene expression was significantly reduced in UC patients vs. CD. *CARD9* protein expression was detected in inflammatory infiltrates; *CARD14* in parenchymal cells, while CARD15 in inflammatory and parenchymal cells. *CARD9*-, *CARD14*- and *CARD15*-expressing cells were observed significantly higher in patients with active UC vs. non-inflamed controls. Therefore the *CARD* family looks indisputably involved in the inflammatory process and might be elaborated in the IBD etiopathophysiology.

There are about 71 CD and 47 UC susceptibility loci/genes to date. Approximately one-third of loci described confer susceptibility to both CD and UC. Amongst these are multiple genes involved in IL23/Th17 signaling (*IL23R*, *IL12B*, *JAK2*, *TYK2* and *STAT3*), *IL10*, *IL1R2*, *REL*, *CARD9*, *NKX2.3*, *ICOSLG*, *PRDM1*, *SMAD3* and *ORMDL3*. The evolving genetic architecture of IBD has furthered the understanding of disease etiopathogenesis. For CD, defective processing of intracellular bacteria has become a central theme, following gene discoveries in autophagy and innate immunity (associations with *NOD2*, *IRGM*, *ATG16L1* are specific to CD). Genetic evidence has also demonstrated the importance of barrier function to the development of ulcerative colitis (*HNF4A*, *LAMB1*, *CDH1* and *GNA12*). According to Chua et al., 2012, there is a strong association between both inflammatory bowel disease gene 5 (*IBD5*) locus variants but not the *IL23R* gene variant with CD (in the Malaysian population) but the *IBD5* locus variants were highest in Indians, which may explain the increased susceptibility of this particular ethnic group to the disease [74].

### 4.2. Intestinal Microbiota

The symbiotically benefit of the intestinal microbiota to the host’s physiology can be divided into three different group categories—(i) nutrition, (ii) immune development, and (iii) host defense [75]. An inauspicious alteration of the constitutional composition and variety of the gastrointestinal microbiota (dysbiosis) is observed and reported in IBD patients which affects the host immune system functionality and barrier integrity, resulting in chronic inflammation and aberrant immune responses [76]. Studies into host-microbe interactions, involving both innate and acquired/adaptive immune responses, have shown to be of particular interest in understanding the possible etiopathogenesis of IBD [76,77]. Evolutions in sequencing advance technology have triumphed to the groundbreaking findings and characterization of the gut microbiota and its role in health and disease. While an altered microbiome has been described in IBD, whether it is a causative source or an effect of the local intestinal response to cellular injury (inflammation) has yet to be illuminated. Moreover, the bidirectional relationship between the intestinal microbiota and the mucosal immune system (discussed on 4.3) adds to the multifaceted complexity of intestinal homeostasis at large. A better understanding of how host genetics, including *NOD2*, influence immune-microbe interactions and alter susceptibility to IBD is still a challenge endeavor and potentially essential in order to gradually manifest therapeutic and preventative precision measures [77].

When compared patients with IBD to healthy individuals, the decrease of bacteria with anti-inflammatory capacities and the increase of bacteria with inflammatory capacities have been reported [78,79]. The most consistent observations are a downsizing in the diversity of gut microbiota and pruning abundance of Formicates [78,80–82]. Expansion in abundance of Proteobacteria and Bacteroidetes have been outlined [78], but downsizing have also been outlined [82]. It has been communicated that *F. prausnitzii*, *Blautia faecis*, *Roseburia inulinivorans*, *Ruminococcus torgues*, and *Clostridium lavalense* are reduced in cases with CD when collated to healthy subjects [83,84] and that the number of *F. prausnitzii* is correlated with the risk of subsequent relapse of ileal CD following surgery. The defect of colonization of *F. prausnitzii* was noted in UC patients during remission and the recovery of the *F. prausnitzii* population after relapse is seen to be associated with the maintenance of clinical remission [85]. In addition, human peripheral blood mononuclear cells simulated with *F. prausnitzii* induced the production of IL-10 and inhibit the population of inflammatory cytokines, such as IL-12 and IFN-γ [86]. Further, a significant reduction of *Roseburia* spp. was noted in the gut microbiota of healthy individual with a high vulnerable genetic risk for IBD. In contrast, a relative increase in Proteobacteria, mainly *E. coli*, was reported in CD patients, in particular, on mucosa-associated microbiota compared to fecal samples [87–94]. CD-associated *E. coli* with pro-inflammatory properties is adhesion-invasive *E. coli* (AIEC), which was initially isolated from adult CD patients [79]. It has been communicated that the number of AIEC increased in about 38% of patients with active CD compared to only 6% in healthy subjects [95]. The increase of pathogenic bacteria with the ability to adhere to the gut mucosa affects the permeability of the intestine, revamps the diversity and composition of gut microbiota, and induces inflammatory reactions by regulating the expression of inflammatory genes, consequently leading to the causing of intestinal inflammation [96]. Further, fluorescence in situ hybridization analyses have shown an enhanced abundance of mucosa-associated bacteria in IBD [97–99]. This may be caused by the altered ecology and increased volume of mucolytic bacteria, such as *Runinococcus gnavas* and *Ruminococcus torques* in IBD patients [99].

The subsequent yield of metabolites affected by the disruption of gut microbiota is reported attributable to the etiopathogenesis of IBD [84]. For example, the concentration of SCFAs has been communicated to decrease in IBD patients, as a result of butyrate-producing bacteria, such as *F. prausnizzi* and *Clostridium* clusters IV, XIVa, XVIII [84]. The reduced production of SCFAs affects the differentiation and expansion of Treg cells and the growth of mucosal cells [100], which play an important part in conserving intestinal homeostasis. On the other hand, the number of sulfate-lessening bacteria, such as *Desulfovibrio*, is abundantly increased in IBD patients [101,102], stemming in the fabrication of hydrogen-sulfate that cause severe injure to the intestinal epithelial cells and induces mucosal damage and inflammation [101]. Collectively, these data forcefully demonstrate that dysbiosis partly is associated with the etiopathogenesis of IBD.

### 4.3. The Intestinal Epithelium and Microbiota

**Figure 1.**
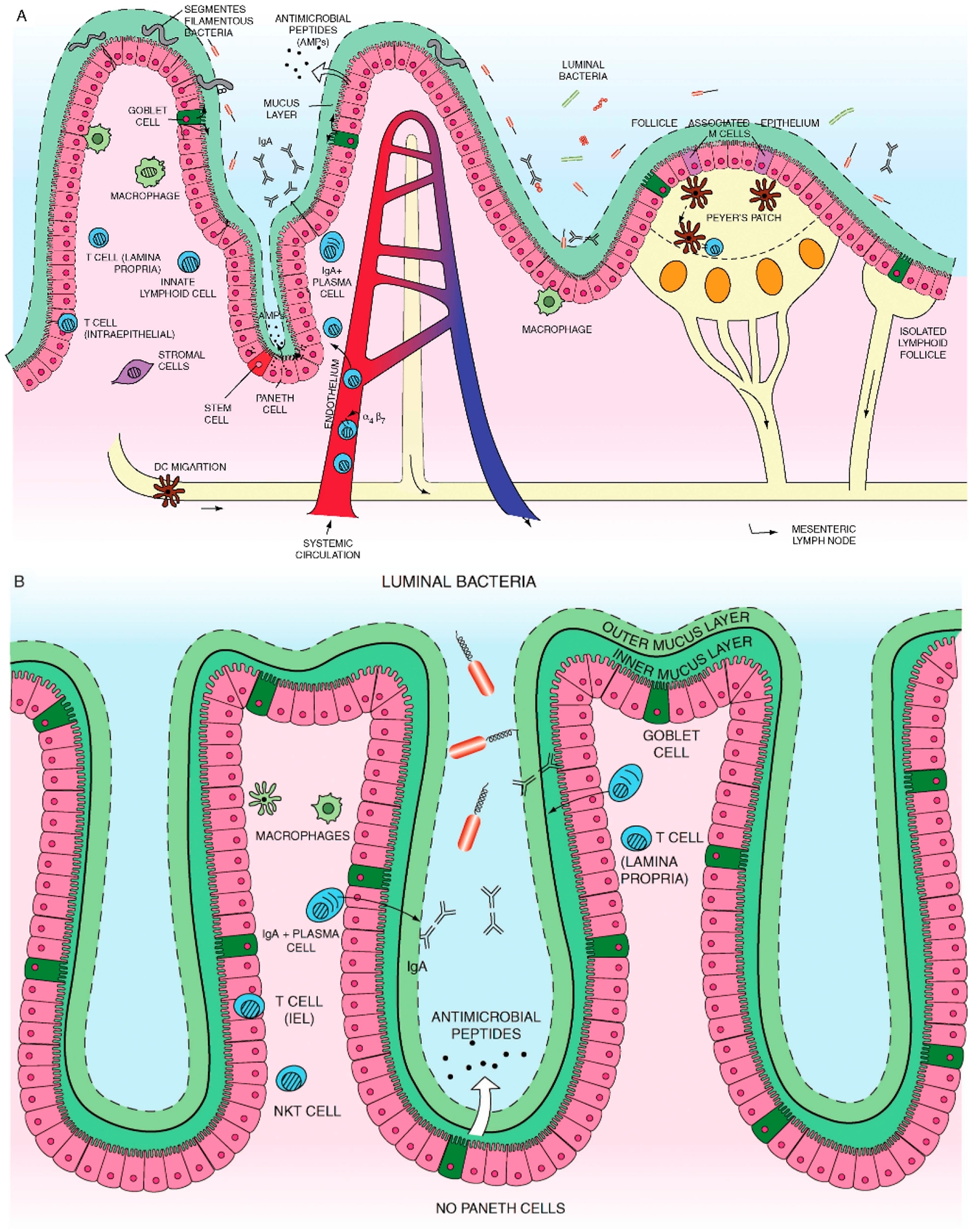
(**A**) Small intestine mucosal immune system landscape. The intestinal epithelial cell (IEC) layers form villi and crypt structures and are composed of different cell lineages. Goblet cells secrete mucus. Paneth cells, found only in the small intestine, can be found at the base of the crypts and are the main secretors of antimicrobial peptides. The base of the crypts also contains the IEC stem cell populations. Immune cells can be found in organized tissue such as Peyer’s patches and throughout the lamina propria. They include macrophages, dendritic cells, intra-epithelial lymphocytes, lamina propria effector T cells, IgA secreting plasma cells, innate lymphoid cells and stromal cells such as fibroblasts. Antigen presenting cells in Peyer’s patches or mesenteric lymph nodes interact with and activate local lymphocytes, which consequently upregulate expression of the integrin α4β7. Such cells then enter the systemic circulation but home towards the gut, in response to chemokine ligands such as CCL25. (**B**) Colon (large intestine) mucosal immune system. The colon has a much higher bacterial load and a markedly different immune cell composition. The colon contains only crypts, no villi. Also there are no Paneth cells, which mean that enterocytes have a much more important contribution to antimicrobial peptide production. However, there is a high prevalence of goblet cells. The mucus forms dual layers, with a thick largely sterile inner layer and a thinner outer layer. There are no Peyer’s patches. While the immune cell types present are similar to those found in the small intestine it is likely that there may be at least subtle differences. In particular natural killer T cells are found more frequently and have a more significant role in the colon. Adapted with permission from “Recent advances in inflammatory bowel disease: Mucosal immune cells in intestinal inflammation” by Cader and Kaser, *Gut* **2013**, *62*, 1653–1664, BMJ Publishing Group Limited [48].

A number of different cells, including enterocytes, goblet cells, neuro-endocrine cells, Paneth cells, M cells, and epithelia resident intestinal stem cells together make the intestinal epithelial cell (IEC) compartment, Figure 1. These monolayer cells structurally self-possessed crypt and villi, with a single columnar cell inner surface with an impervious jointure secreting anti-microbial peptides accommodated mucus; these cells separate intra-luminal pathogens from the sub-epithelial lamina propria [48,103,104].

Normally, there are roughly 10^11^~10^14^ enteric commensal microorganisms from 300~500 different bacterial types [105,106]. These indigenous commensal bacteria play an important duty in defending intestinal homeostasis which has essential impact crucial to nutrient provision, development of the immune system, and regulation of energy metabolism [49,107]. Under certain acquired circumstances these microorganisms can become harmful and can cause intestinal inflammation [108]. Iatrogenically, when patients are treated with a systemic antibiotic drug(s) two or even three times the indigenous/commensal intestinal microbiota get lost and should rebuild to normalize and that could take months. In a compromised luminal innate immune system mechanisms there are some indications that commensal bacteria play crucial role in the developmental trigger of IBD. These include, (i) empiric antibiotic therapy experiences has been satisfying in certain IBD patients [109], (ii) IBD patients have enhanced concentrations against indigenous commensal bacteria [110], (iii) genetic deviants that are consociated with bacterial spotting, such as *NOD2* [111], and T cell immunity, such as IL23R, are incriminated in IBD [112] and (iv) most animal model studies of colitis require commensal bacteria for the initiation or trigger of intestinal inflammation [113]. In addition, recent observations have concentrated on the benefaction of other enteric microorganism, such as viruses or fungi, for IBD elaboration [114,115].

Intriguing observation on stem cell regenerative enrichment report by Marlicz et al. [116]. They observed that developmentally early cells, including hematopoietic stem progenitor cells (HSPCs), mesenchymal stem cells (MSCs), endothelial progenitor cells (EPCs), and very small embryonic-like stem cells (VSELs), were observed mobilized into circulating peripheral blood (PB) in CD patients possibly in response to intestinal tissue injury [116,117]. The mobilized cells also expressed at the mRNA level genes playing a role in development and regeneration of gastrointestinal epithelium accompanied by increased serum concentrations of VEGF and HGF. Therefore it was concluded that CD triggers the mobilization of MSCs, EPCs, and VSELs, while the significance and precise role of these mobilized cells in repair of damaged intestine is still obscure and requires further studies.

#### 4.3.1. Escherichia coli (AIEC)

A number of pathogens have been reported as possible causative microorganisms for IBD establishment trigger. Current studies reveal Proteobacteria, especially adherent-invasive Escherichia coli (AIEC), as one of the candidates. AIEC has been more frequently recognized in patients diagnosed with CD as compared to control subjects [88,90,118]. AIEC is known to be able to capture epithelium and clone within macrophage [119]. Some studies removed AIEC from the small intestine of patients with CD (Crohn’s ileitis) [82,120]. Interestingly, AIEC was infrequently seen in the colon tissue of CD (Crohn’s colitis) patients and was not recognized in UC patients [95], meaning that AIEC performs a vital role in the event of inflammation [121].

#### 4.3.2. Clostridium

Clostridium cluster XIVa and IV are crucial part of gut homeostasis through Treg cell accumulation which is in contrast to AIEC, [122]. Foxp3^+^CD4^+^ Tregs are plenteous in the lamina propria of the large intestine and are crucial immune-regulating cells [123]. Studies revealed that Treg cells were significantly contrived by ileal microbiota [124]. In particular, Treg cells stimulated by Cbir1, a microbiota flagellin, induce IgA + B cells in the intestine. As a result, reduced pathogenic loading by IgA leads to down-regulation of systemic Tcell activation [125]. A development murine quintessential study with an escalated Clostridium XIVa/IV population was observed to be resistant to allergy and intestinal inflammation [122]. Contrastingly, patients with IBD demonstrated a decreased Clostridium XIVa/IV compared to that in non-IBD controls [78,86,126].

**Figure 2.**
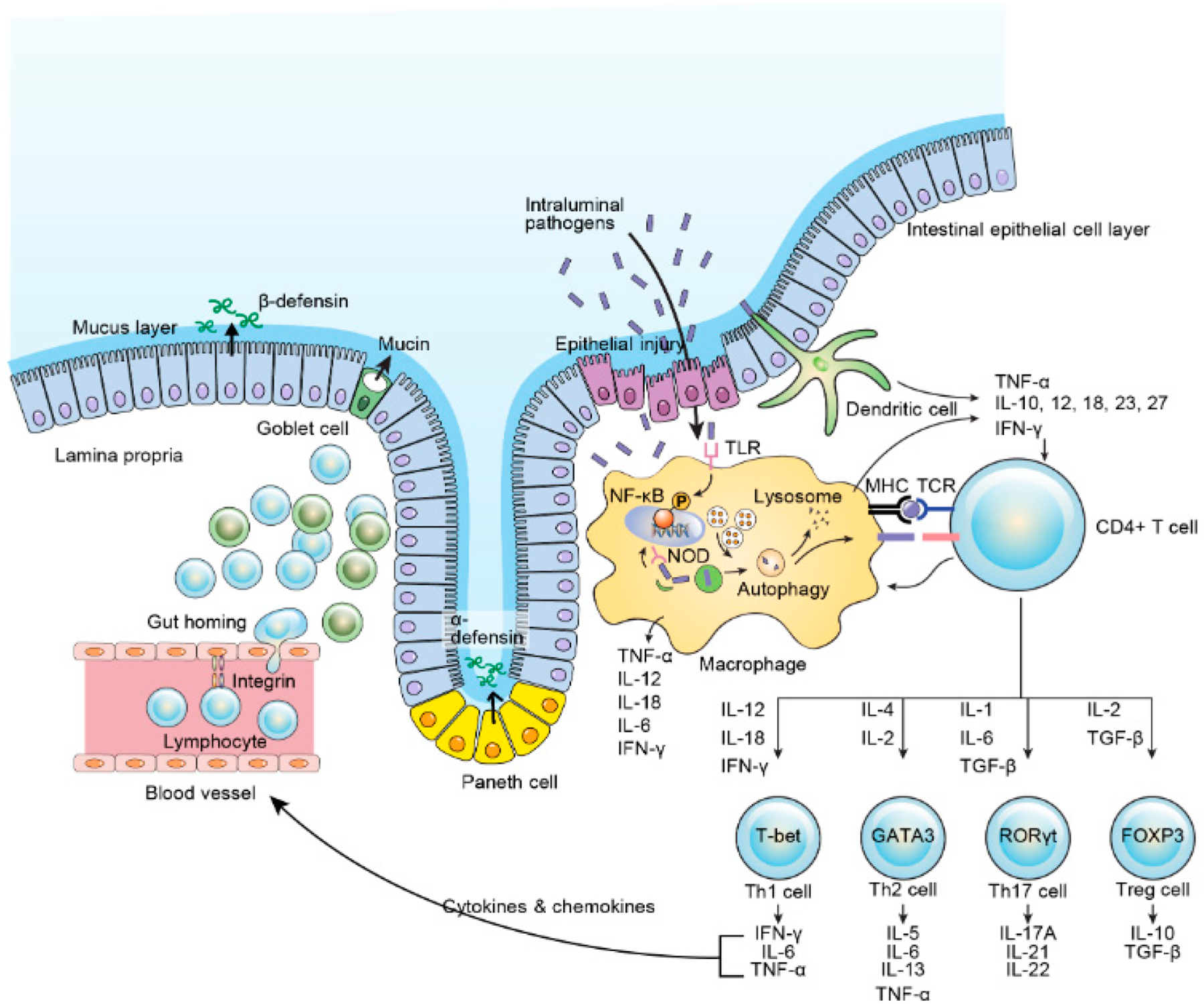
Intestinal immune system. IL, interleukin; IFN, interferon; TNF, tumor necrosis factor; TGF, transforming growth factor; Th, helper T cell; Treg, regulatory T cell; TCR, T cell receptor; NF-κB, nuclear factor kappa-light-chain-enhancer of activated B cell; TLR, toll-like receptor; NOD, nucleotide oligomerization domain. Adapted with permission from “Pathogenesis of Inflammatory Bowel Disease and Recent Advances in Biologic Therapies” by Kim and Cheon, *Immune Netw*. **2017**, *17*, 25–40 [46].

**Figure 3.**
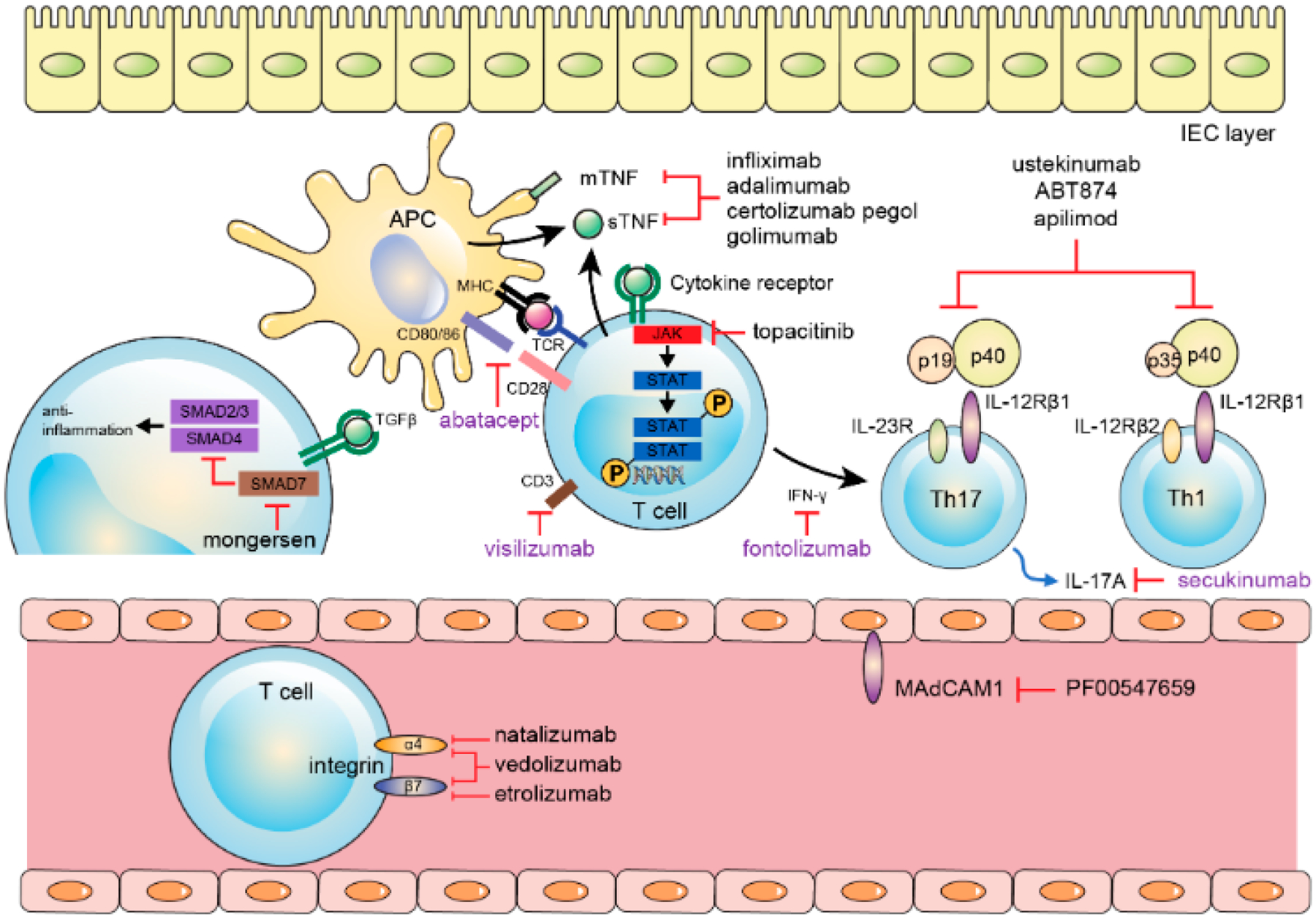
Biologics regarding therapeutic targets (black: showed benefits; violet: no benefits). APC, antigen presenting cell; IEC, intestinal epithelial cell; TNF, tumor necrosis factor; MHC, major histocompatibility complex; TCR, T cell receptor; JAK, Janus kinase; TGF, transforming growth factor; IL, interleukin; MAdCAM, mucosal vascular addressing cell adhesion molecule. Adapted with permission from “Pathogenesis of Inflammatory Bowel Disease and Recent Advances in Biologic Therapies” by Kim and Cheon, *Immune Netw*. **2017**, *17*, 25–40 [46].

Observational studies of dysbiosis in IBD [127] and the disparities in host-microbe relationships which play part to the extent, severity, and chronicity of intestinal inflammation led to efforts to restore microbiota to a normal composition [128]. Further, a successful novel management approach in patients suffering from IBD using fecal microbial transplantation (FMT) has been introduced [129,130]. One randomized control trial involving 75 UC cases demonstrated a significantly escalated remission rate (24%) in individuals cases treated with FMT from unrelated/discrete donor enemas than that in the placebo array (5%) [131]. In another randomized control trial with 48 UC cases yielded antagonistic negative result [132]. To date, there are no randomized control trials contrasting FMT with placebo management in CD population. However, a meta-analysis using four patient series data in 38 CD patients unveiled a 60.5% pooled result rate [133]. It looks likely their outcome was not that of epithelial remission but of clinical response. Consequently, the effectiveness of FMT as a therapeutic use for IBD is still preliminary. Furthermore, optimal donor selection, delivery methods, and donor feces processing, which are both critically important, have not yet been formalized and remain unsettled to date. Probiotics are nutritional supplements that contain microorganisms that when consumed or administered in the proper amount restores beneficial bacteria to the digestive tract and benefit the host’s health. There has been trials made to manage IBD patients by improving intestinal microbial balance through probiotics. In a pilot colitis model study, probiotics demonstrated an anti-inflammatory outcome via TLR9 signaling [134]. In a recent meta-analysis study using 23 randomized controlled trials demonstrated that administering of probiotics was seen to be associated with benefits concerning induction and continuance of remission in patients suffering from UC but painstakingly not in cases suffering from CD [135]. Obviously, these studies are vindicated to draw a concrete conclusion in terms of the management sequels of probiotics in IBD. As IBD-related research advances, expounding of IBD pathologies is accentuating and some of such advances are illustrated in Figure 2. With the opening of the era of biologic and biosimilar agents, it has become realizable to anticipate deep sustainable remission in IBD patients, unalike in the erstwhile; however, about one-third of sufferers painstakingly still do not demonstrate clinical benefit to these modern agents. There are different new biologic and biosimilar agents specific to IBD etiopathogenesis that are now surfacing and are under different phases of clinical trials, Figure 3. With this advancement, more and more patients will likely benefit from these new unfamiliar agents. Moreover, future IBD clinical settings should be used in terms of patient-customized management, and it is expected greatly to shed light clinical practice to have a feasible drug repertoire targeting different mechanisms of the disorder.

### 4.4. Environmental Factors

An environmental factor is an identifiable element in the cultural, demographic, physical, economic, technological environment, or political, regulatory that impacts the operations, survival and growth of an organization and/or institution in health and in disease [136]. IBD is believed to result from several interactions between genetic susceptibility and environmental agents that affect the normal intestinal indigenous/commensal flora to activate an inappropriate mucosal immune response [137]. Despite the fact that IBD susceptibility genes have been elucidated [138,139], similar developments in outlining environmental risk factors have lagged [140,141]. Numerous environmental risk factors have been investigated, including smoking, appendicitis, nutrition, cultural influences on diet, breastfeeding, infections/vaccinations, oral contraceptives, antibiotics, helminths, psychological stress, urban life style, air pollution and childhood hygiene, all portray shared vulnerabilities that could constitute risk for IBD [142–147]. Most of these factors construe the evident relationship between Westernization-urbanization culture and the risk of IBD trigger, as has been described in China and offspring of South Asian immigrants to the United States and Canada [10] and/or United Kingdom [148,149]. These surveillances again raise the issue of how vivid culture influences, such as diet, regulating and/or adjusting the risk of IBD in certain ethnic communities and in certain geographic regions. It will be meaningful to identify the role of these environmental influences/factors in IBD etiopathogenesis triggers [150].

Smoking has been reported to be associated with IBD risk, specifically CD [151,152] and is also reported to be associated with increased intestinal permeability [153], but what remains vague is whether the influence is mediated through the gut microbiome [154]. It also not known whether secondary smoke exposure can increase risk of IBD onset. A meta-analysis study did not identify a relationship between childhood passive-smoke vulnerability and CD [155]; although, additional recent studies have opposite observational results, thus the influence of secondary smoke on IBD assault warrants further investigational studies [156–158].

In humans, psychological stress has been observed to play a significant role in the etiopathogenesis of IBD due to the chronic, relapsing, and remitting nature of this condition [146,147]. The commonly seen chronic and acute stress in these pathologies do alter immune function [142]. Results from experimental studies have been conflicting, with observations endorsing both positive and null connections [146,147]. However, due to the retrospective nature of these studies, recall bias may have influenced the results [142]. Evidence from animal model studies demonstrates that chronic psychological stress may exacerbate IBD by upgrading damage to the gut luminal epithelium, thereby disrupting barrier function [159,160].

Clearly, the environmental risk factors that have been recognized have not nailed down the etiopathogenesis of the “Colitides” to date [161]. These factors mentioned herewith, have been implicated in the increased global incidence of IBD [161,162]. However, even the most conflictingly substantiated environmental risk factor such as smoking is seen to contribute only partially to disease etiopathogenesis. We now know that most people with smoking habits do not have CD and most patients diagnosed with CD are not smokers [161,162]. Agreeably thus, more studies are warranted to better elucidate the environmental determinants of IBD [161,162].

### 4.5. Immune Response

In Colitides, the immune defense against intestinal microbes’ compromises in two different levels [163]: (i) the impairment of epithelial mucosal barrier and (ii) the altered innate and adaptive/acquired host immune responses. The immunopathogenesis of IBD may occur in three distinct stages [163]: (i) penetration of luminal inner contents into amenable tissues which may be facilitated by environmental components such as inherent defects in epithelial barrier or infection, (ii) defective secretion of pro-inflammatory cytokines by macrophages due to compromised clearance of foreign materials from the gut wall and (iii) a compensatory acquired/adaptive immune reaction which results to a chronic inflammatory reaction and gives rise to distinctive IBD abrasions. Briefly, chronic improper activation of the acquired immune system against indigenous commensal microorganism has been observed to be the main etiopathogenesis of IBD [46]. During the process there is increase secretion of IFN-γ from Th1 cells and cytokines associated with Th17 cell, such as IL-17A/F, IL-21, IL-22, and CXCL8, are seen in the intestine of CD cases, while T cells from the lamina propria of UC patients’ significantly produce Th2 cell-associated cytokines, such as IL-5 and IL-13 [48,164,165].

Recently, IL-9-secreting Th9 cells are known to be involved in the pathogenesis of IBD [166]. However, the role of Th9 cells and their secretory cytokine IL-9 in IBD is poorly elucidated and studies on its functional importance in IBD are underway. Clearly, studying the actual role and mechanisms of different T helper cell subsets including Th9 cells in IBD is critical to develop novel IBD therapies. An understanding of the mechanisms that employed by Th9 cells and IL-9 to cause IBD could help contemplate potential targets for the treatment of Th9 cell-mediated IBD.

Conservatively, immune-modulating management of IBD have aligned on acquired/adaptive immunity [167–169]. The NOD2 gene was the first susceptibility gene established within the IBD 1 locus for CD. Subsequently, over 230 genetic risk loci have been identified with IBD and yet NOD2 remains the most robust/powerful association to date [77].

### 4.6. Urbanization

Urbanization is a multidimensional undertaking that manifests as rapidly changing population characteristics and land cover [170]. The rapid urbanization has been observed to correlate with an increasing incidence and prevalence of IBD [4,14]. In the past six decades, IBD has recognized as a rapidly evolving challenge in previously low incidence countries, especially in recently urbanize-industrialized nations, including Africa, Asia, the Middle East and South America [4,16,171–175]. The emergence of IBD in these countries, which are undergoing rapid modernization and urbanization, resembles patterns that were seen in the Western world during the early 20th century, with the upsizing prevalence of UC preceding that of CD in urban areas [10,176]. A meta-analysis of 40 multipronged studies investigating the association between urban environment and IBD observed that the pooled incidence rate ratios (IRRs) for urban vs. rural environments were 1.17 (95% CI 1.03–1.32) and 1.42 (95% CI 1.26–1.60) for UC and CD, respectively [177]. This suggests a bond between urbanization and the incidence of IBD. The relative risk in the urban areas is reported to be 1.3 *versus* countryside [177]. The effects of swift urbanization are likely to be reflected in the human intestinal microbiome that we herewith discussed earlier (Section 4.2), and alterations in the gut microbiome have been reported to be associated with IBD incidences [178]. The role of the gut bacterial microbiome in human health and diseases has been herewith extensively presented [179,180]. Additional studies have outlined that inhabitants residing in non-Western and/or rural areas have a higher bacterial diversity when compared with populations in the Western nations such as United States, Canada and Europe [181–184]. The fecal microbiota of children from a rural African villages, e.g., of Burkina Faso, who mostly eat a diet high in fibre [185], is similar to that of the microbiome of early human settlement at the time of the birth [181]. Children from Burkina Faso demonstrate a significant (*p* < 0.001) enhancement of Bacteroidetes and a reduction of Formicates compared with children from the urban locations, e.g., of Florence, Italy, with a unique plenteous of bacteria from the genera *Prevotella* and *Xylanibacter*, which are recgnized to contain a set of bacterial genes for cellulose and xylan hydrolysis. These bacteria were totally lacking in the 15 European country children studied [181]. Corresponding observations have been seen in children and adults in Malawi, Amazonian American Indians [183] and adult Hadza hunter-gatherers in mainland Tanzania [182]. *The Hadza* of *Tanzania, in Eastern Africa*, despite human civilization, are one of the very few societies in the world who still live by hunting and gathering [182]. These studies have shown that urbanization is consociated with an upsizing proportion of *Bacteroides*, *Alistipes* (Bacteroidetes), *Balautia*, *Faecalibacterium*, *Ruminococcus* (Formicates) *Bifidobacterium* (Actinobacteria) and *Bilophila* (Proteobacteria), whereas Prevotella (Bacteroidetes) is significantly elevated in the gut microbiota of individuals residing in non-industrialized communities [181–183].

Studies comparing the rural and urban microbiome within a population of homogeneous ethnicity is a scarcity [185]. In one study comparing the fecal microbiota composition of African descendants residing in rural, semi-urban communities with those settling in urban locations, substantial dissimilarities were observed, with *Prevotella* predominating in semi-urban individuals and *Bacteroides* predominating in urban African Americans [186]. These observations indicate that the intestinal microbiota content contrasts between genetically similar populations living in diverse communities, such as rural vs. urban. Comparison of the fecal microbiota of elderly persons residing in rural and urban areas in Japan demonstarted that individuals living in Yuzurihara (a rural village) had a larger number of bifidobacteria, whereas larger proportions of bacilli and lecithinase-positive clostridia were found in residents of Tokyo [187]. An underway countrywide study of IBD incidence in Asia shows that Inner Mongolia has a low IBD incidence than other regions [185]. Microbial profiling of Inner Mongolia residents indicates that the high-level presence of *Phascolarctobacterium*, *Lactobacillus* and *Bifidobacterium* might be associated to a pasturing lifestyle and a diary diet. *Lactobacillus helvetucus* is oftentimes found in individuals from every rural pasturing area in Inner Mongolia but not in Mongolians living in Hohhot city (urban), indicating that diet affects the intestine luminal microbial composition of Mongolians [188]. A Russian study [189] demonstrated that microbial communities from inhabitants in rural areas had a 2.6-fold increase in the frequency of new microbial community structures distinct from the common three enterotypes [190] compared with the microbial communities of urban hosts. The predominant microbial populations in rural inhabitants were from the Formicates and Actinobacteria phyla. These bacterial communities are accommodated by the consumption of starch-rich bread and potatoes, typical staple foods utilized in rural Russia, and natural foods that are available to low-income socioeconomic communities from their household gardens [189,191]. Correspondent with the theory of vanishing microbiota and its relation with the emergence of autoimmune and chronic GI disorders [192], the constantly witnessed loss of microbiota affluent and diversity during urbanization might largely account for the upsizing in IBD incidence. These experimental and observational studies were mostly based on 16S ribosomal RNA (rRNA) gene sequencing [185]. All-in-all, an in-depth understanding of rural vs. urban intestinal bacterial species or strains and their functions in IBD etiopathogenesis is largely still lacking warranting more experimental studies.

Indisputably, an air contamination is taking place in parallel with urbanization [170,193], and it is thought to have significant detrimental effect on a wide range of public health issues, including IBD [143,194]. Persistently long-term exposure to a high concentration of nitrogen dioxide pollution and particulate matters has been reported to be related with an escalated risk of early-onset of CD, indicating a linear increase proportional to high concentrations of pollutants [143]. Other forms of inhaled environmental exposures observed to promote the susceptibility to IBD via alteration of the intestinal microbiota are herewith in-depth discussed [195–220].

### 4.7. Dietary/Nutrition

Intestinal mucosal microbiota composition is dependently influenced and modified by diet [221,222]. According to the IBD-EPIC (IBD European Prospective Cohort) Study, several dietary factors have been found to be related with IBD onset [223–226]. Milk and milk product consumption was observed to be potentially related with a low risk of developing CD (*p* = 0.23) but not for with UC (*p* = 0.60) [224], whereas a role of flavones (C_15_H_10_O_2_) and resveratrol (C_14_H_12_O_3_) in the risk of triggering CD was also shown [225]. The IBD-EPIC study results also indicate dietary role for linoleic acid in the etiopathogenesis of UC [225]. According to this calculated study cohort, overweight, as measured by BMI (body mass index), was observed not consociated with the etiopathogenesis of either UC or CD, respectively [226]. Altogether, total fiber consumption from vegetables, cereals or fruits, and the ensuing evolution of either CD or UC were found not consociated [223]. Nonetheless, it is acknowledged that most of these studies are grounded on food intake frequentness questionnaire data that may have significant boundaries/limitations [227]. There is an indisputable evidence indicating a role for diet, particularly among genetically susceptible individuals and the development of IBD [44]. In addition, data from animal model studies demonstrate that dietary change and/or modification impacts the risk of IBD [44]. More studies report to have seen affirmation suggesting that artificial sweeteners such as saccharin, sucralose, acesulfame potassium (ace K) and cyclamate may have played causative role in the pathogenesis of IBD [228–230]. These sweeteners are observed to be distinctive by high stability with little metabolism by the body and long-lasting in the environment but high inhibitory effect on bacteria [231]. In one EPIC (European Prospective Investigation into cancer and Nutrition) demonstrated that a dietary pattern obtaining of high intake of sugar and sweetened beverages and low consumption of vegetables is linked with elevated hazard of UC (incidence rate ratios for the fifth vs. first quintile, 1.68 [1.00–2.82]; *P*trend = 0.02) [228]. In contrast, a recent sizable prospective cohort study from Scandinavian demonstrated no corroborations between consumption of sweetened beverage and subsequent endangered CD or UC [232]. The study established 143 incident cases of CD (incidence rate = 11 cases/100,000 persons-years) and 349 incidence cases of UC (incidence rate = 28 cases/100,000 person-years) over 1,264,345 person-years of follow up. Consumption of sweetened beverages does not appear to increase endanger of CD (*P*trend = 0.34) or UC (*P*trend = 0.40) [232]. Compared to participants who reported no consumption of sweetened beverage, the calculated multivariable-adjusted HRs for 1 or more consumptions per day were 1.02 for CD (95% CI, 0.60–1.73) and 1.14 for UC (95% CI, 0.83–157), respectively. The association between consumption of sugar-sweetened beverages and risk of CD or UC were not modified by age, sex (cohort), body mass index, or smoking (all *P*interaction ≥ 0.12) [232].

Gene-environmental studies on the interrelationship between environmental factors and genetic variant in functionally annotated genes have largely been encouraged to assist infer causal affiliation [233] to shed light into potential biological mechanistic pathways through which an environmental component such as diet might shed light to the aetiopathogenesis of IBD. Genetic loci related with IBD risk can be widely designated into those involving the innate and/or acquired immune response anomalies and mucosal barrier functional event [234]. Experimental studies also show that a number of these pathways are impacted by nutritional/dietary factors [235]. Thus, it is biologically creditable that special and/or specific dietary ingredients have unique differential outcomes on the incidence of IBD, coinciding with the genetic background of every individual per sig.

Introductory analyses of gene-environmental interplay in studies involving dietary factors in IBD have been increasingly promising [236–239]. A study from the NHS and NHSII, which involved 169 patients with CD and 202 cases with UC matched to 740 participants as control, analyzed the reciprocal action between total dietary consumption of iron and heam iron and genetic variants related with risk factors of IBD [240]. This analysis illustrated a relationship between iron and haem iron consumption and the UC vulnerability locus rs1801274, a coding variant in the *ECGR2A* (which encodes the low affection immunoglobulin-γ Fc location receptor IIa) gene. Peculiarly, among females with the GG genotype, low haem iron consumption was linked with a consequentially reduced risk of UC (or 0.11, 95% CI 0.03–0.37 for each 1g increase in heam iron consumption. In contrast, increasing haem iron consumption was correlated with an almost threefold elevated risk of UC among females with the TT genotype (OR 2.26, 95% CI 1.02–7.48). Owing to the important role of FCGR2A in controlling humoral reaction to infection [241] and the known importance of the rs1801274 variant in changing the binding volume capacity of the encoded protein product of C-reactive protein (CRP) and immunoglobulin G2 (IgG2) [242–244], these results offer supportive attestation for an interesting interplay between dietary haem consumption and immune physiological function in the etiopathogenesis of UC.

In an identical population, in a NCC (nested case-control) study of 202 cases with UC and 169 patients with CD in the NHS and NHSII cohorts matched to 740 participants as control based on age, menopausal situation, period of blood withdrawal collection and fasting status, an interplay between dietary potassium consumption and genetic variants in the IL-23 pathway that have been previously related with risk of IBD in GWAS was identified [234,236]. Particularly, the rs7657746 variant of *IL21* (which encodes IL-21) appeared to modify the relationship between potassium consumption and risk of IBD pathogenesis. Each additional 200 mg more in dietary potassium consumption was inversely consociated with risk of UC (OR 0.90, 95% CI 0.82–0.98) among participants with the AA genotype, but not among those with the AG or GG genotypes. Similar observations were reported in cases with CD in the study. As IL-21 play a key developmental role of T_H_17 cells through signal transducer and driver of transcription 3 (STAT3), a transcription factor needed for the differentiation of T_H_17 cells in vivo, the results from gene-environment interaction studies indicate an existence of a potential mechanism for the identified relationship [245]. IL-21 and IL-23 induced expression of the nuclear receptor RORγ (also known as RORC), which, in synergy with STAT3, upstairs IL-17 expression in CD4^+^ T cells, leading to the activation of T_H_17 cells [245]. In addition, IL-21 hinders the transforming growth factors-β (TGFβ)-dependent generation of FOXP3^+^ Treg cells and catalyzes T_H_17 cells activation [246]. Intriguingly, the gen-environment interplay finding was encouraged further supported by in vitro studies showing that potassium generates FOXP3 expression in naïve and memory T cells and in pro-inflammatory T_H_17 cells. This effect was noted even in the presence of pro-inflammatory cytokine, indicating that potassium curbs inflammation in a pro-inflammatory milieu.

Chassaing et al., reported in animal model that, relatively list content of emulsifiers, such as carboxymethylcellulose and polysorbate-80, caused low-grade inflammation and obesity/metabolic syndrome in WT hosts and incubated robust colitis in mice predisposed to this gastrointestinal disorder [247]. Emulsifier-induced metabolic syndrome was associated with microbiota encroachment, altered species composition, and increased pro-inflammatory potential. Use of germ-free mice and fecal transplants demonstrated that such alterations in microbiota were essential and enough for both low-grade inflammation and metabolic syndrome. These observations support the emerging idea that perturbed host-microbiota associations resulting in low-grade inflammation can increase adiposity and its associated metabolic effects. Moreover, they suggest that broad use of emulsifying agents might be contributing to increased societal incidence of obesity/metabolic syndrome and other chronic inflammatory diseases such as IBD.

Finally, studies from the NHS and NHSII have vividly indicated that two variants in CYP4F3, which encodes the cytochrome P40 4F3 enzyme (CYP4F3) elaborated in PUFA metabolism, might modify the relation between n-3 and n-6 PUFA intake and risk of UC [238]. Potentially, the relation between n-3:n-6 PUFA intake ratio and UC was modified by rs4646904 single nucleotide polymorphism (SNP) in CYP4F3 (*P*interaction = 0.049). A high (greater than or equal to the medium) n-3:n-6 PUFA intake ratio was related with a lower risk of UC among women with the GG or AG genotypes (OR 0.57, 95% CI 0.32–0.99), but not among those with the AA genotype (OR 0.95, 95% CI 0.47–193). Identical observations were also reported earlier in a pediatric case-control study with newly diagnosed CD [239], suggesting that the interaction is indeed robust.

### 4.8. Role of miRNA in IBD trigger

Advances in the field of miRNA (microRNA) research technology is speedily expanding [246] and are strongly associated in the etiopathogenesis of IBD, having an important role in the development, regulation and differentiation of both the innate and acquired/adaptive immune system [248]. A number of studies have demonstrated a differential expression of miRNA in tissue and blood samples from cases suffering from IBD compared with normal/healthy controls, indicating that miRNAs may be shortlisted not only in the development of immune system component but as new candidate biomarkers of these disorders [249]. Due to the fact that CD and UC differ in their clinical presentations, genetic consociations, gene expression patterns, and immune reactions, differing miRNA profiles are anticipated for these two IBD pathologies. It is now been realized that CD and UC patients have unique miRNA expression profiles in their target organs. Not surprising that while some uniquely expressed miRNA are commonly routine to other immune-related disorders, most are different. Further, studies have demonstrated peculiar miRNA expression profile bio-fingerprints in IBD and preliminary operational analyses relate these deregulated miRNA to canonical pathways related with IBD etiopathogenesis [250]. In order to elucidate precision roles of miRNAs is the human context more studies are required despite current promising to advance understanding of miRNAs in the pathogenesis and diagnosis of IBD which may be useful for the development of miRNA-based therapies [251].

In recent years, blood-derived microparticle biochemical peptides have become invaluable for IBD monitoring and more frequently used as surrogate markers of intestinal inflammation. Emerging concepts that revolve around measurement of cell-derived microvesicles (MVs) in the circulating blood vascular bed of IBD patients is another emerging advances advantageous in future disease understanding and management. Extracellular microvesicles (ExMVs) are part of the cell secretome baroque in chronic autoimmune diseases, such as IBD. ExMVs capture functional RNA species and proteins from one cell to another, an observation that paved up the new way to the new field of research of bioactive molecules in cell-to-cell communications [252–254]. This observation disclosed up the gates to novel idea, in which the presence of mRNA, noncoding RNA, and miRNA in ExMVs in blood and other biological body fluids gave the possibility of employing ExMVs as new fingerprint biomarkers for pathological disorders. Subsequently, ExMVs has become a target for “liquid biopsy” strategies. Tziatzios et al. [255] observed that circulating levels of platelet derived microparticles (PDMPs) were enhanced in CD patients but did not correlate with disease activity. 5-ASA treatment was associated with lower levels of PDMPs, while anti-TNF-α treatment did not influence expression of ExMVs in IBD patients. Similarly, circulating PDMPs were increased in IBD patients with active disease.

## 5. Clinical Diagnosis

Currently, “Diagnostic Gold Standard” test for IBD does not existent. Also, there is no accurate tools to predict whether a patient with newly diagnosed IBD will progress to complications of disease before and after colectomy for UC or indeterminate colitis such as cancer, pouchits, cufittis or fistulae, structuring and penetrating complications in de novo CD. In the IBD clinical setting, clinicians use state-of-the-art criteria, yet engage in invasive and inexact testing classification systems such as endoscopy (gastroscopy and colonoscopy), radiologic imaging, and histopathology to diagnose IBD patients, resulting to a substantial number of incorrect or delayed diagnosis [256–260]. It is possible that early accurate diagnosis and timely treatment could improve outcomes for those high-risk patients [13]. Even with a combination of recommended state-of-the-art diagnostic system modalities IBD patients cannot be accurately diagnosed. Up to 15% of colonic IBD cases are classified as “indeterminate colitis (IC)” because the established criteria for UC and CC are non-definitive [257,261,262]. In addition, another 15% of colonic IBD cases that are prescribed pouch surgery (restorative proctocolectomy (RPC) with ileal pouch-anal anastomosis (IPAA), which are standard surgical procedures for treating UC or IC predicted as UC, are in fact CC cases. Therefore, a total of 30% of colonic IBD patients are not diagnosed accurately [258–260]. For these reasons, efforts at identifying accurate, noninvasive biomarkers have been undertaken [263–265]. Another really challenge is a significant subgroup of IBD patients, especially UC patients undergoing proctocolectomy that convert to *de novo* Crohn’s, are thought to be “misdiagnosed” [265]. This may not be accurate because there is a possibility that these patients with UC were “transformed” due to an altered microbiome ecology in the pouch and immune environment and that the patient actually “convert”. Since there is significant overlap between disease-associated genes, it is possible that disease phenotype may change within a given individual. More elucidation studies are needed in this area.

A recent breakthrough finding that Paneth cell specific peptide “Human alpha-defensin 5 (DEFA5)” delineate colonic IBD (CC versus UC) may solve diagnostic dilemma in IBD clinical settings [265]. Detection of DEFA5 more accurately circumvented the IC cases into UC or CC phenotype and identified CC cases initially treated as UC cases [265]. Among patients with IC, DEFA5 is a reliable delineator with a positive predictive value of 96 percent [265]. The distinction between UC and CC is of utmost importance when prescribing a patient’s candidacy for pouch surgery, RPC and IPAA [40,266,267].

## 6. Management and Challenges

As discussed on Section 4.3, persons suffering from IBD are frequent users of the healthcare system, with an annual frequency of hospitalization exceeding 20 percent [32]. Studies from the United States economic implications report of IBD, showed in 2014, that CD and UC were related with annual direct and indirect costs ranging between US $14.6 and $31.6 billion [268]. Costs include invasive endoscopic and radiologic procedures for diagnostics and management decisions, as well as medications, hospitalizations, and surgical interventions [269]. Further costs accrue to community in loss of productivity and disability of impacted patients with deprived quality of life. While a noninvasive, easier, accurate and fast screening diagnostics tool is needed to downsize costs and burden of disease [265] the unmet need for noninvasive markers has outpaced the evidence. Thus, IBD is indeed expensive to treat and manage [270]. Due to an incomparable infrastructural gape in terms of access to care between developing vs. developed nations and the uneven representation of IBD across socioeconomic strata, a serious plan is required in the developing countries concerning how to tackle this emerging human health challenge [4,137].

There are significant advances in genetic and immunologic analytical technologies and as a result new therapeutic approached are now in place that accurately target the mechanistic pathways of IBD [46]. Apart from conventional immune-suppressive treatment, the evolution of biological and biosimilar agents that are target specific has lead in more frequent and deeper remission in the IBD patients, with mucosal healing as a therapeutic goal. In not too distant future, targeted novel biologic and biosimilar agents should defeat the obstacles of customary treatments and ensure that each patient can be managed with optimal medications that are nontoxic and precisely target IBD. These drugs are unfortunately the quickest-cultivating partition of the prescription drug market in the West as is IBD in the developing countries [4]. The healthcare system, and certainly the patients, in developing nations will struggle and will not be able to afford such costly managements which will lead into a series of events that are life threatening in terms of health, safety or well-being of large group of people.

Fecal microbiota transplantation (FMT) is emerging advances as a novel approach to therapy for UC. However, the interpretation of efficacy of FMT for UC is disputably complicated based on various study contentions, FMT administration procedures, intensity of therapy (dose) and donor stool processing methodologies. In a systemic review with meta-analysis including randomized controlled trials (RCTs), Costello et al. reported that despite variation in stool processes, FMT appears to be effective for induction of remission in UC patients, with no major short-term safety signals [271]. However, further elucidative observational studies are required to better define, establish, and verify dose frequency and preparation methods, and to explore its feasibility, efficacy and safety as a maintenance agent.

## Abbreviations

IBD: inflammatory bowel Disease
UC: ulcerative colitis
CD: Crohn’s disease
MOOSE: meta-analyses of observational studies
PRISMA-P: preferred reporting items for systematic review and meta-analysis protocols
MEDLINE: Medical Literature Analysis and retrieval system online
EMBASE: Excerpta Medica dataBASE
CINAHL: Cumulative index of Nursing and Allied Health Literature
SCFAs: short-chain fatty acids
NOD2: Nucleotide-binding oligomerization domain-containing protein 2
TNF-α: tumor necrosis factor-alpha
